# Endosaccular Coil Embolization of Ruptured Anterior Inferior Cerebellar Artery Pseudoaneurysm After Gamma Knife Surgery for Vestibular Schwannoma: A Case Report and Literature Review

**DOI:** 10.3390/jcm13216595

**Published:** 2024-11-02

**Authors:** Byung Hyun Baek, Seul Kee Kim, Yun Young Lee, Hyoung Ook Kim, You Sub Kim, Sung Pil Joo, Woong Yoon

**Affiliations:** 1 Department of Radiology, Chonnam National University Medical School, Chonnam National University Hospital, Gwangju 61469, Republic of Korea; yunyoung0219@gmail.com (Y.Y.L.); cnurad232@jnu.ac.kr (H.O.K.); 2 Department of Radiology, Chonnam National University Medical School, Chonnam National University Hwasun Hospital, Hwasun 58128, Republic of Korea; kimsk.rad@gmail.com; 3 Department of Neurosurgery, Chonnam National University Medical School, Chonnam National University Hospital, Gwangju 61469, Republic of Korea; yskim22@jnu.ac.kr (Y.S.K.); nsjsp@jnu.ac.kr (S.P.J.)

**Keywords:** pseudoaneurysm, endosaccular embolization, vestibular schwannoma, gamma knife surgery

## Abstract

**Background:** Ruptured pseudoaneurysm of the distal anterior inferior cerebellar artery (AICA) in patients with a history of gamma knife surgery (GKS) for vestibular schwannoma (VS) is rare. Several previous reports have described treatment strategies for radiation-induced pseudoaneurysm in the AICA: either surgical trapping or endovascular parent artery occlusion of the AICA. **Methods:** We present the first case of endosaccular coil embolization for a ruptured pseudoaneurysm in a large-diameter AICA after GKS for VS, successfully preserving the parent AICA. **Results:** Major recanalization of the coiled pseudoaneurysm was observed on follow-up imaging 3 months after the initial endovascular treatment. The patient subsequently underwent additional endosaccular coil embolizations for regrowth of the treated pseudoaneurysm buried in the VS. Two years later, another major recanalization was detected, prompting further retreatment. Subsequently, the patient has remained in a stable condition for 4 years. **Conclusions:** We suggest that endosaccular coil embolization of the distal AICA aneurysm with parent artery preservation might be a safe and feasible treatment option for radiation-induced saccular pseudoaneurysm arising from a large parent artery. However, close and regular follow-up imaging and preparation for potential retreatment are necessary, as pseudoaneurysms coiled within VSs are prone to recanalization.

## 1. Introduction

Distal anterior inferior cerebellar artery (AICA) aneurysms are rare, and one known cause of distal AICA aneurysms is stereotactic gamma knife surgery (GKS). These aneurysms are considered pseudoaneurysms based on pathologic findings and carry a high risk of rupture. Several previous reports have described ruptured pseudoaneurysm of the distal AICA in patients with a history of GKS for vestibular schwannoma (VS) [1,2,3,4,5,6,7,8,9,10]. Three cases were treated with surgical trapping [3,4,9]. Seven cases were treated by endovascular parent artery occlusion (PAO) of the AICA with coils or n-butyl cyanoacrylate (n-BCA) [1,5,6,7,8,9,10]. These treatments carried inevitable ischemic complications in almost all cases. Due to the adhesive nature of these AICA pseudoaneurysms, treatment has typically involved trapping or PAO. However, when the AICA is responsible for critical perfusion of the cerebellum, alternative treatment methods must be considered. Umekawa et al. [11] reported a case of a large-diameter, unruptured AICA pseudoaneurysm that was successfully treated with surgical trapping combined with an occipital artery–AICA bypass to preserve distal AICA perfusion. We present a case of a ruptured pseudoaneurysm arising from a large-diameter AICA in a patient with a history of GKS for VS, successfully treated with endosaccular coil embolization. This marks the first instance of this treatment approach among previously reported cases, with the parent artery being preserved. Further, we discuss the pathomechanism of the pseudoaneurysm and the clinical course of the patient following endosaccular coil embolization, along with a literature review.

## 2. Case Presentation

**Figure 1:**
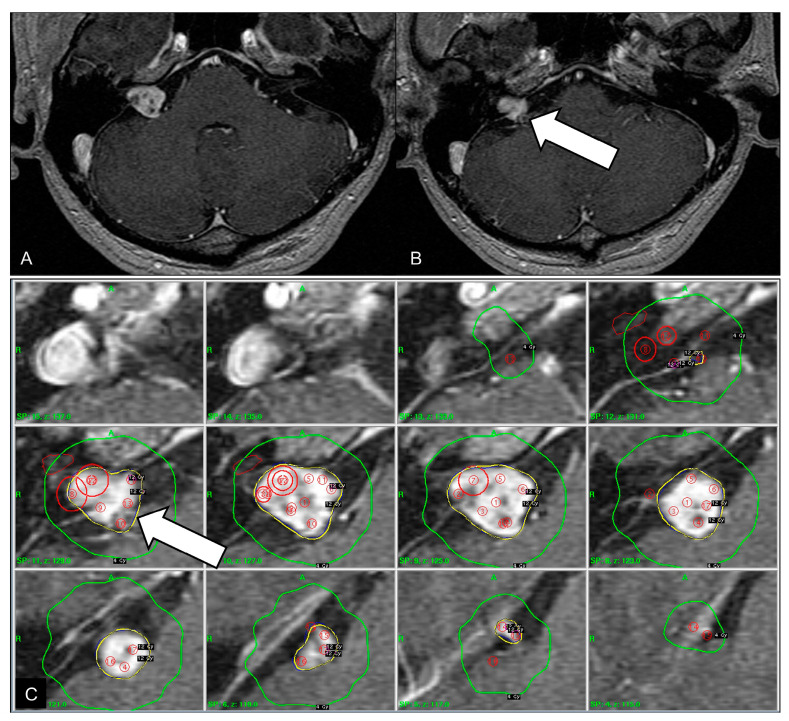
Initial magnetic resonance images before gamma knife surgery. (**A**) Contrast-enhanced axial T1-weighted image showing a 1.5-cm vestibular schwannoma with internal necrotic content. (**B**) The inferior part of the vestibular schwannoma is in proximity to the neighboring anterior inferior cerebellar artery (arrow). (**C**) Axial dose distribution targeting the tumor, based on contrast-enhanced magnetic resonance imaging. The 12 Gy isodense line is outlined in yellow and the 4 Gy isodense line is outlined in green. The 12 Gy isodense curve encompasses the anterior inferior cerebellar artery (arrow).

A 42-year-old man presented with a sudden onset of mental deepening. Five years ago, the patient underwent GKS for VS (Figure 1). The tumor margin was covered by the 50% isodense line, and 12 Gy was delivered to the margin. The isodense curves targeting the inferior part of the tumor compromised the neighboring AICA (Figure 1C). This vascular observation was overlooked at that time because time-of-flight magnetic resonance (TOF MR) angiography was not performed prior to GKS. He had right hearing impairment before GKS. Serial magnetic resonance images demonstrated partial regression of the tumor after GKS and did not show any vascular disorder of the neighboring AICA.

**Figure 2:**
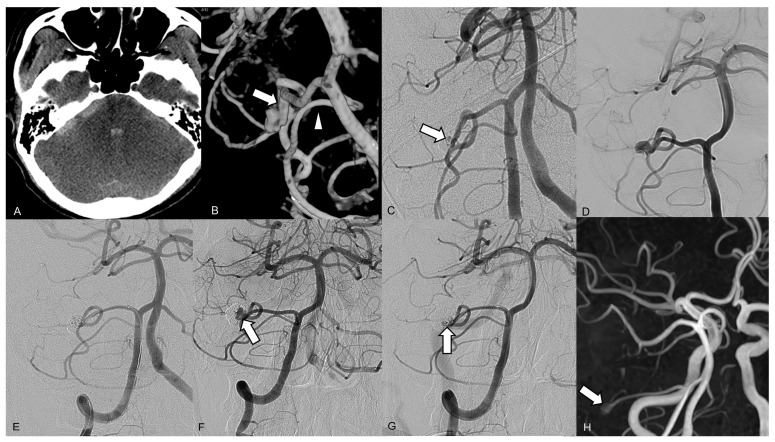
Brain images from a 42-year-old male who presented with a sudden onset of mental deepening. (**A**) The axial brain computed tomography scan on admission shows a subarachnoid hemorrhage, predominantly distributed in the right cerebellopontine angle cistern, and a small intraventricular hemorrhage. (**B**) A three-dimensional reconstruction of the right vertebral artery angiogram reveals a lobulated wide-neck aneurysm arising from the right distal anterior inferior cerebellar artery (AICA) within a meatal segment. The right AICA (arrow) was larger than the posterior inferior cerebellar artery (arrowhead). (**C**) The right vertebral artery angiogram performed immediately after the endosaccular embolization shows a tiny remnant portion of the neck (arrow). (**D**) The 3-month follow-up angiograph shows major recanalization at the superior part of the coiled aneurysm. (**E**) The final angiogram after the second coil embolization shows complete occlusion of the pseudoaneurysm, with preserved distal AICA flow. (**F**) The 2-year follow-up angiograph shows major recanalization (arrow) at the base of the coiled pseudoaneurysm. (**G**) The final angiogram after the third coil embolization shows near complete occlusion of the pseudoaneurysm, with a tiny remnant lesion (arrow) at the base. (**H**) Follow-up magnetic resonance angiography, 4 years after the final embolization, shows a small remnant sac (arrow) at the base of the coiled pseudoaneurysm.

On admission computed tomography (CT), a diffuse subarachnoid hemorrhage was revealed with a predominance of distribution in the right cerebellopontine angle cistern, and a small intraventricular hemorrhage was noted (Figure 2A). Brain CT angiography showed a small contrast pooling near the tumor without a definite connection to the regional vascular structure. Diagnostic catheter angiography revealed a lobulated wide-neck aneurysm arising from the right distal AICA at a meatal segment (Figure 2B). The aneurysm was located at a nonbranching site. A robust right AICA, with a diameter larger than that of the ipsilateral posterior inferior cerebellar artery (PICA), stemmed from the right AICA–PICA complex. Subsequently, endovascular therapy was attempted under general anesthesia. After positioning a 6 Fr guiding catheter in the right vertebral artery, we placed a Headway 17 microcatheter (MicroVention, Aliso Viejo, CA, USA) with a preshaped tip angle of 90° into the aneurysmal lumen over a microwire. Endosaccular coil embolization was performed using five coils, successfully preserving the parent artery and leaving a tiny remnant portion of the neck (Figure 2C). The patient was discharged without any neurologic deficits. Follow-up TOF MR angiography and conventional angiography 3 months after the endovascular procedure revealed recanalization of the coiled pseudoaneurysm, with regrowth of the aneurysm sac from the residual neck (Figure 2D). The second endosaccular coil embolization was performed in the same manner, resulting in complete occlusion of the recurred sac on final angiography (Figure 2E). Thereafter, retreatment was performed due to recanalization of the pseudoaneurysm 30 months after the first embolization (Figure 2F,G). No ischemic complications occurred after either endovascular treatment. Since the final embolization, the patient has been undergoing TOF MR angiography check-ups annually, and a 3-mm small remnant sac has remained stable without changes for 4 years (Figure 2H).

## 3. Discussion

Radiation-induced vasculopathy can affect both small and large intracranial arteries, and pseudoaneurysms can develop in previous radiation fields. To date, eleven cases of ruptured pseudoaneurysm in the AICA after GKS for VS have been reported [1,2,3,4,5,6,7,8,9,10]. The lateral pontine segment of the AICA is divided into premeatal, meatal, and postmeatal segments, according to the relationship with the porus of the internal acoustic meatus [12]. In the present case, the pseudoaneurysm occurred in the meatal segment, while previously reported cases of AICA pseudoaneurysms have occurred in the premeatal or meatal segments, which are considered to be within the radiation field (Table 1). The time interval between GKS and rupture of the pseudoaneurysm in our case was 4 years and 5 months, the shortest reported in the literature to date. A retrospective review of the GKS planning images revealed that the AICA was within the radiation field, which likely contributed to the development of the pseudoaneurysm in our case. This might also explain why the rupture occurred in the shortest time following GKS among reported cases of pseudoaneurysms. It is likely that the AICA was overlooked prior to GKS because TOF MR angiography was not performed. Therefore, it is essential to conduct thin-slice CT angiography or magnetic resonance angiography before GKS treatment of VS to assess the proximity of a potentially large AICA. The mechanism of pseudoaneurysm formation in the AICA after GKS was documented as a radiation injury to the parent artery vessel wall and subsequent dissection [5]. This theory was in line with pathologic findings of surgically resected pseudoaneurysms in two previous reports [3,4], in which there was no residual elastic lamina in the aneurysm wall and a thin collagenous wall of the aneurysm with loss of elastica and media. In addition, an intraoperative finding of a radiation-induced pseudoaneurysm of the AICA documented that the aneurysm wall was unified with the tumor membrane, and the aneurysm was buried by a tumor [3,4].

The optimal treatment strategy for patients with ruptured pseudoaneurysm of the AICA after GKS for VS has not been established. Decisions might be influenced by the diameter of the parent artery harboring the pseudoaneurysm, and by anatomic features of the pseudoaneurysm, such as location, shape, and accessibility. Three cases were treated with surgical trapping of the aneurysms [3,4,9]. Seven cases were treated by endovascular PAO of the AICA with either coils or n-BCA [1,5,6,7,8,9,10]. These treatments carried inevitable ischemic complications such as facial palsy, abducent palsy, ataxia, dysarthria, bulbar palsy, and cerebellar and brachium pontis stroke [1,4,5,6,7,8,9,10], although two cases treated with either surgical trapping or PAO did not describe postoperative status (Table 1). Park et al. [2] reported self-resolution of pseudoaneurysm after the failure of coil embolization. To our knowledge, this is the first case of endosaccular coil embolization for ruptured pseudoaneurysm of the AICA after GKS for VS with preservation of the parent artery. In our case, the pseudoaneurysm arose from a robust AICA, which had a larger diameter than the ipsilateral PICA. In all previously reported cases of AICA, pseudoaneurysms presented with ruptured status, and the diameters of the parent artery were small compared to the present case. Endovascular PAO of a large AICA, as in the present case, might result in a territorial stroke. As a treatment method that preserves the large AICA, in addition to endosaccular coiling, surgical trapping, or PAO of the AICA, along with distal AICA–occipital artery bypass surgery, could be considered. Umekawa et al. [11] reported a case of an unruptured AICA pseudoaneurysm that was successfully treated with surgical trapping and an occipital artery–AICA bypass. A radiation-induced pseudoaneurysm developed in the AICA, which perfused a large area of the cerebellum and was larger than the ipsilateral PICA following GKS for VS. In the present case, the saccular form of the ruptured pseudoaneurysm on conventional angiography was considered more suitable for endosaccular coiling rather than trapping and bypass. Therefore, we performed endosaccular coil embolization to prevent ischemic complications.

In the present case, the patient underwent additional coil embolization twice for recurrent regrowth of the coiled pseudoaneurysm. It is considered that vulnerability to recanalization after coil embolization might be explained by the relationship between the pseudoaneurysm and VS. This speculation is based on the pathologic features of two previous reports of surgically treated pseudoaneurysm of the AICA after GKS related to VS: Akamatsu et al. [3] reported that the pseudoaneurysm was buried in the VS, and the aneurysm had only a thin collagenous wall on pathologic examination; Yamaguchi et al. [4] described unification of the aneurysm wall with the tumor membrane and a lack of elastic laminae on histologic examination. Further, based on previous reports with pathologic examination of radiation-induced aneurysms that documented macrophage infiltration in the aneurysms, macrophages may be a culprit for the degradation of adjacent tumor membranes. Thus, we propose that the high probability of recanalization might be attributable to the vacuum phenomenon, resulting from the lack of a pseudoaneurysm wall layer and degradation of the enclosing VS due to radiation injuries. Hemodynamic stress from the large AICA towards the pseudoaneurysm may also contribute to recanalization. Despite achieving satisfactory embolization in our case, the two instances of major recanalization can be explained by degradation and hemodynamic stress. After the final embolization, the coiled pseudoaneurysm and the surrounding tumor are considered to have stabilized.

The adequacy of endosaccular embolization for radiation-induced pseudoaneurysm might be debatable, and the fate of coiled pseudoaneurysm is still difficult to predict. However, given the nature of pseudoaneurysms prone to recanalization, short-term follow-up is necessary after endovascular coil embolization. In the present case, major recanalization was detected only 3 months after the first endosaccular coil embolization and another major recanalization occurred 2 years later, leading to further retreatment. Matsumoto et al. [13] reported a recurrence 1 year after endovascular coil embolization for a radiation-induced aneurysm that developed in the distal internal carotid artery following GKS for a pineal germinoma, which required retreatment. They explained that there was a higher risk of recanalization of radiation-induced pseudoaneurysm after coil embolization because radiation-induced de novo aneurysm is formed by regional vasculopathy and is not a focal injury; the researchers also emphasized the importance of close follow-up after endovascular treatment.

Re-rupture of pseudoaneurysms is another major concern after endovascular therapy because pseudoaneurysms have fragile walls. Choi et al. [14] reported a case of a dissecting aneurysm of a distal AICA aneurysm at the meatal loop treated with intra-aneurysmal embolization with parent artery preservation, leaving a small residual portion of the aneurysm neck. Subsequently, the patient experienced recurrent hemorrhage from the recanalized aneurysm 1 month after embolization. The patient was treated with occlusion of the parent artery, just proximal to the aneurysm. Uchikawa et al. [15] reported two cases of pseudoaneurysm presenting with subarachnoid hemorrhage after GKS for trigeminal neuralgia: one patient died after re-rupture of the pseudoaneurysm before intervention. Therefore, treatment of radiation-induced pseudoaneurysm should be planned immediately, and endovascular therapy can be attempted.

Only a few papers have reported AICA aneurysms treated with endosaccular coil embolization [16,17,18,19], and these cases involved aneurysms that were not induced by radiation. Although endosaccular embolization of a distal AICA aneurysm might yield ischemic complications, Oh et al. [16] reported a case of a ruptured distal AICA aneurysm beyond the meatal loop that was successfully treated, and AICA flow was preserved. However, unilateral hearing loss occurred, which the authors explained as possibly caused by temporal occlusion of the AICA by the microcatheter. In the present case, the patient had already experienced right hearing impairment due to ipsilateral VS before GKS. In our case, the patient already had hearing impairment, and no additional ischemic complications occurred after endovascular treatment. However, based on previous reports, even when an AICA aneurysm is treated with endosaccular embolization while preserving the parent artery, unilateral hearing impairment can still occur.

## 4. Conclusions

We report a case of ruptured pseudoaneurysm in the AICA after GKS for VS. Recurrent regrowth from the coiled pseudoaneurysm embedded in the VS necessitated further retreatment. Endosaccular coil embolization may be a safe and feasible treatment option for pseudoaneurysm arising from a large parent artery. However, close and regular follow-up imaging is necessary to monitor for early recanalization, as radiation-induced pseudoaneurysms are prone to recanalization, as highlighted in the present report.

## Figures and Tables

**Table 1:** Summary of cases of ruptured pseudoaneurysm of the anterior inferior cerebellar artery after gamma knife surgery for vestibular schwannoma.

No.	Author (Year)	Age/Sex (at GKS)	Marginal Dose (Gy)	Interval from GKS to Rupture	Site of Pseudoaneurysm	Treatment	Ischemic Complications
1	Takao et al. (2006) [1]	63/F	12	6	Premeatal	Endovascular PAO with coils	Facial palsy
2	Park et al. (2009) [2]	69/F	12	5	Premeatal	Failed EVT	Not applicable
3	Akamatsu et al. (2009) [3]	75/F	12	8	Meatal	Surgical trapping	ND
4	Yamaguchi et al. (2009) [4]	67/F	25	6	Meatal	Surgical trapping	Facial palsy
5	Sunderland at al. (2014) [5]	50/F	13 + 12	10	Premeatal	Endovascular PAO with coils	Dysarthria, bulbar palsy
6	Mascitelli et al. (2015) [6]	59/M	ND	6	Premeatal	Endovascular PAO with n-BCA	Cerebellar and brachium pontis infarction
7	Matsumura et al. (2015) [7]	49/F	ND	15	Meatal	Endovascular PAO with coils	Hydrocephalus
8	Matsumura et al. (2015) [7]	29/F	ND	16	Meatal	Endovascular PAO with coils	ND
9	Murakami et al. (2016) [8]	61/M	18	12	Premeatal	Endovascular PAO with coils	Facial and abducens palsy, ataxia
10	Takahashi et al. (2023) [9]	66/M	12	28	Meatal	Surgical trapping	Facial paralysis
11	Wakuta el al. (2024) [10]	68/M	20	20	Meatal	Endovascular PAO with coils and n-BCA	Pontine infarction, facial palsy
12	Present case	37/M	12	4	Meatal	Endosaccular occlusion with coils	None

Abbreviations: EVT, endovascular treatment; F, female; GKS, gamma knife surgery; M, male; n-BCA, n-butyl cyanoacrylate; ND, not described; PAO, parent artery occlusion.

## Data Availability

The data that support the findings of this study are available from the corresponding author, upon reasonable request.
